# Optoelectronic array of photodiodes integrated with RRAMs for energy-efficient in-sensor computing

**DOI:** 10.1038/s41377-025-01743-y

**Published:** 2025-01-15

**Authors:** Wen Pan, Lai Wang, Jianshi Tang, Heyi Huang, Zhibiao Hao, Changzheng Sun, Bing Xiong, Jian Wang, Yanjun Han, Hongtao Li, Lin Gan, Yi Luo

**Affiliations:** 1 Department of Electronic Engineering, Tsinghua University, Beijing, China; 2 Beijing National Research Center for Information Science and Technology (BNRist), Tsinghua University, Beijing, 100084 China; 3 School of Integrated Circuits, Tsinghua University, Beijing, 100084 China

**Keywords:** Imaging and sensing, Photonic devices

## Abstract

The rapid development of internet of things (IoT) urgently needs edge miniaturized computing devices with high efficiency and low-power consumption. In-sensor computing has emerged as a promising technology to enable in-situ data processing within the sensor array. Here, we report an optoelectronic array for in-sensor computing by integrating photodiodes (PDs) with resistive random-access memories (RRAMs). The PD-RRAM unit cell exhibits reconfigurable optoelectronic output and photo-responsivity by programming RRAMs into different resistance states. Furthermore, a 3 × 3 PD-RRAM array is fabricated to demonstrate optical image recognition, achieving a universal architecture with ultralow latency and low power consumption. This study highlights the great potential of the PD-RRAM optoelectronic array as an energy-efficient in-sensor computing primitive for future IoT applications.

## Introduction

With the widespread use of artificial intelligence (AI) and 5 G network^1^, the Internet of Things (IoT) is developing rapidly. The growing data generated by the IoT has brought a burden to the data center (even though breakthroughs in in-memory computing for electrical and optical domains^2–5^), which requires new technologies for terminal devices. Edge computing devices^6^ can be applied to processing visual, auditory, tactile, and other signals to provide efficient processing and reduce data transmission^7^. Emerging “sensing with computing” on-chip architectures^8^, including near- or in-sensor computing^9^, make a difference for terminal devices that operate on the IoT. Compared to traditional off-chip processing in Von Neumann architecture, image processing in front-end sensors reduces communication and computation burdens, thereby improving time and energy efficiency.

So far, a variety of architectures have been developed to implement image processing functions^10^ (including contrast enhancement, noise suppression, recognition, etc.). The main forms and characteristics of existing in-sensor computing are summarized in Table 1. Some studies have implemented near- or in-sensor computing through circuit designs^7^, where processing elements (PEs) and circuits are made near or within the pixel array, respectively^11–15^. Also, Sony^16^ proposes a 3D integrated vision chip to vertically integrate the functional layers (sensor, memory, computing, communication, etc.) in space, with a processing speed reaching 1000 frames per second (fps). Apart from circuit designs, technologies involving novel material systems and advanced devices have recently been adopted. The memristor array enables parallel in-memory computing on the output from the photoelectric sensor array^17–22^, thus simplifying PE circuits. Besides, some photonic synaptic devices made of detect-and-memorize (DAM) materials have been proposed for in-sensor computing, exhibiting multi-level conductance states and long-term plasticity (LTP) under various light conditions^6,23–34^. In addition, optoelectronic sensors that integrate sensing, storage, and processing functions have attracted much attention due to acting as an artificial neural network (ANN) for IoT applications^1,10,35–37^. In short, to better deliver the potential of the IoT, multifunctional integrated devices and low-power consumption systems are desired^1^.

**Table 1:** Overview of the main forms and characteristics of existing in-sensor computing

Ref.	Architecture design	Application	Operation speed	Power consumption	Wavelength band
^12^	CMOS SIMD image processors (ALU)	Edge detection	4.6 ms for integration and 60 μs for processing	Power-supplied	Visible
^14^	A CMOS hybrid architecture that integrates an image sensor, three processors, and a neural network	Pattern recognition	~1 ms per operation	Power-supplied (630 mW@50 MHz)	Visible
^16^	Vision Chip with 3D-Stacked Column-Parallel PEs	Spatial processing, multi-target tracking	~1 ms per operation @0.31 Mpixels 4b	Power-supplied (363 mW@0.31 Mpixels 4b)	Visible
^17^	RC system with GaO_x_ optical synapses as the input of reservoir and memristor array as readout network	Fingerprint recognition	Performed under optical pulse of 25 ms width	Power-supplied	DUV
^10^	Ferroelectric photosensor network (non-volatile)	Image recognition, edge detection	Response <1 ns	Self-powered (R_e_ ≤ 0.2 mA/W)	UV
^30^	Graphene/MoS_2−x_O_x_/graphene photomemristor (non-volatile)	Feature extraction, image recognition	Response ~1.38 ms	Self-powered (R_e_ ≤ 98.8 mA/W)	Visible
^33^	Two-terminal opto-sensor based on multilayer γ-InSe flake	Visual adaptation behaviors	Fast response ~3.2 s; slow response ~34.1 s	Self-powered (~0.01 nA@5 mW/cm^2^ illumination)	ultraviolet to near-infrared
This work	PD-RRAM array (non-volatile)	Image recognition	Response ~30 ns (@Si PD) with pixel-level parallel computing	Self-powered (*R*_e_ ≤ 0.2 A/W)	Universal

Here, we demonstrate an architecture that integrates photodiodes (PDs) with resistive random-access memories (RRAMs) to implement in-sensor computing for image recognition. Hundreds of silicon PDs integrated with RRAMs are fabricated on a chip, and the PD-RRAM unit cell exhibits multi-level photovoltaic responses as controlled by RRAMs that have non-volatile and multi-resistance state characteristics. These characteristics make the PD-RRAM cell to be a highly reliable and self-powered unit with adjustable photo-responsivity. Multiple individual cells are then wired into the PD-RRAM array, whose capability to perform multiply-accumulate computation (MAC) between optical images and weights is experimentally verified. The PD-RRAM array is further used to implement real-time letter recognition by physical networks with high accuracy. In summary, the architecture is presented to achieve pattern recognition and image pre-processing, which reduces the amount of image data from sources and improves the efficiency of the optoelectronic signal conversion. This type of architecture provides a real-time machine vision approach for the IoT, featuring high integration, ultralow latency, and low power consumption.

## Results

### PDs integrated with RRAMs

**Fig. 1:**
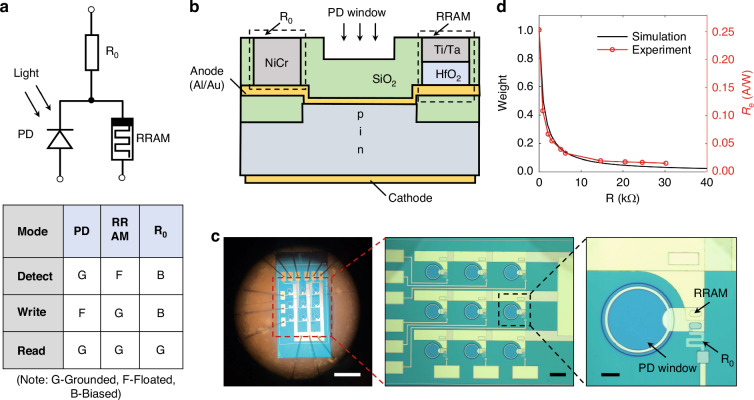
Photodiodes (PD) integrated with RRAMs. **a** Schematic of a single PD-RRAM cell. Detecting mode: control terminals of the R_0_ and PD with the RRAM terminal floated. Writing mode: control terminals of the R_0_ and RRAM with the PD terminal floated. Reading mode: control three terminals under the short-circuit condition. **b** The corresponding schematic of the cross-sectional view of the PD-RRAM cell. **c** Microscope image (left) of chip packaging. Scale bar, 0.6 mm. Microscope image (middle) of the fabricated 3 × 3 PD-RRAM array. Scale bar, 120 μm. Microscope image (right) of the PD-RRAM cell. Scale bar, 30 μm. **d** Resistance-tunable weight (theoretically) and photo-responsivity (experimentally). The weight (that is, terminal current ratio of the RRAM to PD in the reading mode) set by programming RRAMs, is proportional to the *R*_e_ (that is, ratio of the *I*_out_ to the incident light power in the reading mode)

The conventional photovoltaic device (such as p-i-n, n-p-n, etc.) exhibits a constant photo-responsivity (*R*_*e*_), where the photocurrent (*I*_ph_) grows with the power of incident light *P* (i.e., *I*_ph_ = *R*_*e*_ *×* *P*). To implement tunable optoelectronic outputs, we design a unit consisting of a PD used for detecting light, a fixed resistance, and an RRAM used for weight storage. The designed PD-RRAM cell has a three-terminal structure, as schematically illustrated in Fig. 1a. The *R*_*e*_ can be electrically modulated by setting RRAM resistance values. The PD-RRAM cell has three working modes for its application, namely detecting mode, writing mode, and reading mode. For the detecting mode, terminals of the R_0_ and PD are under control with the RRAM terminal floated, and the image information is output by the *I*_ph_ of the PD; for the writing mode, terminals of the R_0_ and RRAM are under control with the PD terminal floated, and the RRAM is set into the target; for the reading mode, the current of the RRAM terminal (*I*_out_) is output under illumination with three terminals grounded.

The schematic of the cross-sectional view is illustrated in Fig. 1b. The PD has a p-i-n structure, and a layer of HfO_2_ film with the metal-insulator-metal (MIM) structure is fabricated on the top of the anode to form RRAMs. Holes for RRAMs are created by etching through a patterned SiO_2_ isolation layer and terminating at the surface of the pad. The high resistivity material (such as NiCr, TiN, etc.) is used as R_0_, sputtered on the oxide. Detailed fabrication steps are described in Methods and Fig. S1. The HfO_2_-based RRAM is chosen because of its light insensitivity, low cost, easy preparation, stable performance, scaling down, and non-volatile properties. This type of design could be universally used for PDs of various materials (such as Si, GaAs, InP, GaSb, etc.). In this work, it’s used to fabricate PD-RRAM cells built on a silicon wafer shown in Fig. 1c. The cells are wired into a 3 × 3-pixel array where each pixel consists of a Si p-i-n detector with a diameter of 120 μm, an RRAM with an area of 200 μm^2^ and a resistance made of NiCr alloy. A circular region is directly exposed to light from above, as a window of the PD. Contact electrodes are arranged on the front and back respectively.

According to the principles of operations, the RRAM resistance (*R*) changes the magnitude of the *I*_out_ for a given power of the incident light, modifying the terminal current ratio of the RRAM to PD under the short-circuit condition (i.e., weight). The *R*_*e*_ (that is, a ratio of the *I*_out_ to the incident light power in the reading mode) is proportional to the weights (*w*) set by RRAM resistance,

1 $$w=\frac{{R}_{0}}{R+{R}_{0}}$$

Taking R_0_ as 1 kΩ, when *R* is changed from 1 kΩ to 100 kΩ, the weight ranges from 0.0099 to 0.5. The number of discrete weights increases with the number of RRAM resistance states. The resistance-tunability of *w* (theoretically) and *R*_*e*_ (experimentally) are shown in Fig. 1d. This dependence is measured with a fixed white LED power of 10 mW/cm^2^, whereas *R* varies from 1 kΩ to 30 kΩ. The measured *R*_*e*_ (Fig. 1d) exhibits the expected modulation and it shows the stability of programming.

### Programmable optoelectronic output

**Fig. 2:**
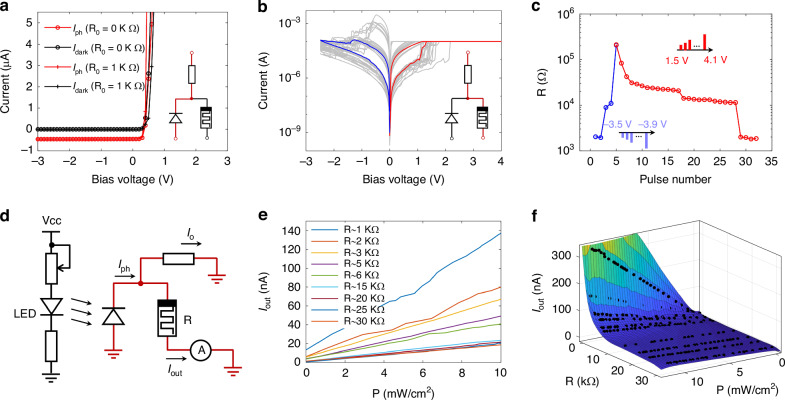
Reconfigurable optoelectronic output of the PD-RRAM cell. **a** Current–voltage characteristics of the PD connected in series with R_0_ in the dark (black line) and under illumination (red line) in the detecting mode. **b** Current–voltage characteristics of the RRAM connected in series with R_0_ under DC sweeping voltage with a SET compliance current (I_CC_) of 100 μA in the writing mode. The gray lines show 20 reliable switching cycles of an individual RRAM. The transited current states during SET (or RESET) indicated by red (or blue) lines are shown. **c** One-cycle linearly modulated depression (blue) and potentiation (red) behaviors as functions of the number of RESET pulses of −3.5 to −3.9 V and SET pulses of 1.5 to 4.1 V for 10 ns. The inset shows varying voltage amplitude results in multi-resistance states for SET and RESET. **d** Illustration of the *I*_out_ when PD-RRAM cells are operated in the reading mode with LED illumination. **e** Dependence of the *I*_out_ with varying resistance states of RRAMs, with LED light intensity increasing from 0 to 10 mW cm^−^^2^. **f** Fitting of the *I*_out_ collected in **e**

The dependence of the optoelectronic output (*I*_out_) on the RRAM resistance (*R*) as well as light power (*P*) is further investigated. Figure 2a shows the detecting mode of the PD-RRAM cell with sweeping voltages under light/dark conditions and Fig. S2b shows the measured spectral response for different wavelength (shown in Fig. S2a). The p-i-n diodes possess a self-powered characteristic under the condition of zero bias, which is almost unaffected by a small load circuit (such as 1 kΩ). In other words, the *R*_*e*_ of PDs does not change with a small load circuit, at zero bias. In this architecture, the PD could be regarded as a current source that changes with light due to its characteristics. The RRAM plays the role of weight storage, with non-volatile data retention and programmable multi-resistance states. Figure 2b shows 20 consecutive direct current (D.C.) write/erase behaviors on switching characteristics of an individual HfO_2_-based RRAM with a compliance current (*I*_CC_) of 100 μA in the SET process and a stop voltage of −2.5 V in the RESET process, and a switching window of ~100 can be obtained with a low resistance state (LRS) of ~1 kΩ and a high resistance state (HRS) of ~100 kΩ. With the typical SET or RESET pulse train applied to the RRAM, the resistance switches from 1 kΩ to 100 kΩ with at least 10 resistance states (as shown in Fig. 2c, Fig. S5a, b). Some works^38–48^ focus on improving the analog switching behaviors of RRAMs, which is considered to be achievable in HfO_2_-based RRAMs. In 2023, Rao et al.^38^ report HfO_x_-based memristors fabricated on chip achieve 2048 conductance levels, which reaches the 11-bit floating-point precision. Here we demonstrate the feasibility of this architecture, instead of optimizing the continuous tunability of RRAMs.

A system is setup to test the programmable output (*I*_out_) of the PD-RRAM cell under white LED illumination in the reading mode, as illustrated in Figs. 2d and S6. The *I*_ph_ generated by the PD is shunted into two dividing currents (i.e., the *I*_out_ and *I*_*o*_) whose magnitudes are proportional to the *I*_ph_. The measured output for varying RRAM resistance states and light intensities is shown in Fig. 2e. As the *P* of LED light increases from 0 to 10 mW/cm^2^, the *I*_out_ increases linearly with the light power that the window of the PD receives. As *R* ranges from 1 kΩ to 30 kΩ, the *I*_out_ increases with the decrease of *R*, which results from the shunt ratio of the circuit. It is according to the theoretical principles, as expected. In order to understand the dependence more intuitively, the surface fitting of the short-circuit output (*I*_out_) in three dimensions is shown in Fig. 2f. According to the side view from left, the *I*_out_ is inversely proportional to *R* at the same light intensity, which means the results of Fig. 1d are consistent across light intensities.

The linear dependence of the *I*_out_ on *P* for any given *R* programmed is confirmed, and the *R*_*e*_ changes with *R* as expected. It verifies the programmable optoelectronic output characteristic of the PD-RRAM cell, which is useful for analog multiplication between *P* and a given *R*_*e*_. The integrated PD-RRAM cell, as demonstrated above, has prospects of constructing the PD-RRAM array with in-sensor computing capability.

### Transient optoelectronic response

**Fig. 3:**
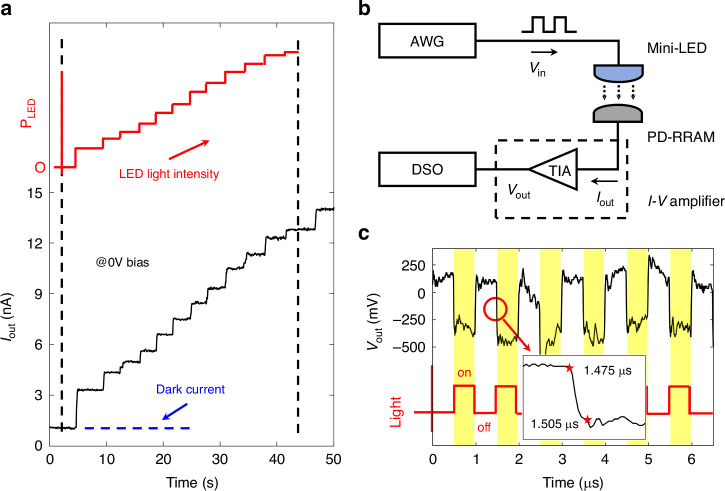
Transient optoelectronic response of the PD-RRAM cell. **a** Measured short-circuit output current time sequence diagram. A white LED is used as the light source, with light intensity (red) ramped up from 0 to 5 mW cm^−^^2^. **b** Schematic illustration of the setup for speed tests. An arbitrary waveform generator (AWG) outputs a 1 MHz square wave signal to a blue Mini-LED, and the *I*_out_ of the PD-RRAM cell is connected to a trans-impedance amplifier (TIA) and converted into a voltage signal to be measured by the oscilloscope. **c** Output response of the PD-RRAM cell with the pulsed light (red line) under the short-circuit condition. The inset shows the rise and fall times of a pulse, measured between 10% and 90% of the full pulse amplitude, which are both 30 ns

The characteristics of the high response speed and low noise are required for the PD-RRAM cell to ensure its real-time image recognition. The test system is setup (Figs. 2d and S6) to measure the transient optoelectronic response. Figure 3a demonstrates the short-circuit output current (*I*_out_) time sequence with the *P* of LED light stepped up from 0 to 5 mW/cm^2^. The PD-RRAM cell has a fast response and responds linearly to light intensity, with a low dark current (~10^−^^9^ A) and little noise (~10^−11^ A). The high signal-to-noise ratio (SNR) is crucial for the physical analog calculation of the photocurrent signals, improving the accuracy of image recognition. The fast response and low noise show the potential for real-time processing of optoelectronic image signals.

Besides, the high-speed response measurement system is setup to get the upper limit of the PD-RRAM cell, as schematically illustrated in Figs. 3b and S7a. A blue mini-LED is adopted as the light source, with the 3 dB bandwidth of ~15 MHz. The mini-LED emits optical switching signals to the PD-RRAM cell, with frequency of 1 MHz. The *I*_out_ of the PD-RRAM cell is amplified into the voltage signal (*V*_out_) measured by the oscilloscope (Figs. 3c and S7c), which exhibits a voltage pulse sequence of 1 MHz. The rise and fall times of a pulse, measured between 10% and 90% of the full pulse amplitude, are both 30 ns under the short-circuit condition, longer than those of an individual PD by 5 ns (more details in Fig. S7b, c). The response speed of PDs degrades slightly with a load circuit (RRAM and R_0_), at zero bias. As the RRAM has a read time of less than nanosecond speed^49^, the response speed of the PD-RRAM cell is mainly limited by the PD response time and RC time constants. The RRAM mainly has an effect on the load R_L_ of PD,

2 $${R}_{L}=\frac{R\times {R}_{0}}{R+{R}_{0}}$$

As we know, the smaller the R_L_, the faster the response of PDs. This design maintains the numerical range of total load R_L_ < R_0_ ~ 1 kΩ, and thus keeps PDs working at a high response speed.

### In-sensor computing as a classifier

**Fig. 4:**
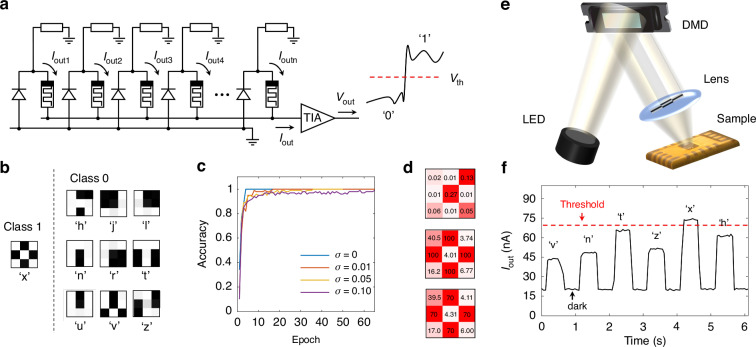
A 3 × 3 PD-RRAM array for in-sensor computing as a classifier. **a** Schematic illustration of the PD-RRAM array for analog MAC operations. The accumulated output current (*I*_out_) is generated by *N* pixels that are connected in parallel. **b** Letter images of 3 × 3 pixels used for training/inference with 2-class. **c** Accuracy of the classifier during training under different noise levels (*σ* represents the variance of image noise). **d** From top to bottom: theoretical dimensionless weights, theoretical RRAM resistance states, actual RRAM resistance states after programming. The unit of resistance is kΩ. **e** Schematic illustration of the optical setup for recognition. The LED light is spatially modulated by a DMD and resulting images are projected onto the PD-RRAM array. **f** Measured output of the PD-RRAM array for recognition. Projection of different letters with a duration of 0.5 s, leads to the distinct *I*_out_

The PD-RRAM array for in-sensor computing is demonstrated, performing analog MAC operations between optical images and weights. And this type of architecture can be used to implement real-time image processing, such as pattern recognition by a single-layer perceptron. As illustrated in Fig. 4a, the PD-RRAM array consists of *N* pixels (i.e., *N* cells), with pixels connected in parallel for analog computing (i.e., inference). An efficient MAC operation between optical images and weights are performed in this array under the short-circuit condition, just like mathematical matrix calculations in neural networks. In this array, the *R*_*e*_ and *w* can be converted accordingly as shown in Fig. 1d. The multiplication of the *R*_*e*_ and *P* occurs at each pixel, and *N* output currents generated by pixels are summed together according to Kirchhoff’s law. The total output current (*I*_out_) is expressed as

3 $${I}_{out}=\mathop{\sum }\limits_{i=1}^{n}{I}_{out\_i}=\mathop{\sum }\limits_{i=1}^{n}{R}_{e\_i}\times {P}_{i}$$

class="removeElement">where *R*_*e_i*_ and *P*_*i*_ are the photo-responsivity and input light power at the *i-th* pixel, respectively. The *I*_out_ is converted to an output voltage (*V*_out_) via a trans-impedance amplifier (TIA), and then the *V*_out_ is fed to a sigmoid activation function to generate a nonlinear output by software or circuits. When the *V*_out_ exceeds the threshold voltage (*V*_*th*_), it is judged to be recognized as 1; Otherwise, it is recognized as 0.

To experimentally verify the image recognition function, a 3 × 3 PD-RRAM array (shown in Fig. 1c) is used for recognizing by real-time processing of optoelectronic signals. In the training process, a set of images of letters with 3 × 3 pixels are used in training/inference (Fig. 4b), and the target letter (i.e., ‘X’) is classified into class 1 while others are class 0. The training of this architecture is first carried out as a classifier, adopting a single-layer perceptron (fully-connected network with 9 inputs, 1 output, 1 layer) with fully positive weights. Figure 4c shows the accuracy of the classifier during training under different noise levels (*σ* represents the variance of image noise), and the accuracy eventually tends to 95%~100% in the back propagation (BP). It verifies the feasibility of this architecture in theory, with certain anti-noise performance. Thus, the corresponding weight matrix is obtained by training, and the RRAM resistance states can be converted accordingly with the weight matrix (as shown in Fig. 1d). Then, the weight matrix is transferred to the PD-RRAM array where RRAMs are programmed into theoretical resistance states. Here, the theoretical weights, theoretical RRAM resistance states and actual RRAM resistance states after programming are shown in Fig. 4d, and the deviations between the actually programmed weights and the theoretical ones are small.

After programming, the PD-RRAM array could directly conduct the inference once an optical image is input. To test it, the optical setup system for image recognition is shown in Figs. 4e and S8a. More specifically, LED light is spatially modulated by a digital micromirror device (DMD) and the resulting images are projected onto the 3 × 3 PD-RRAM array chip. At this point, PDs in the array receive corresponding light intensities (*P*_*i*_) and generate photocurrents (*I*_ph_), which are collected after being diverted by RRAMs as the *I*_out_ according to (3). Thus, the PD-RRAM array would conduct MAC operations, and output results of recognition by the *I*_out_. Figure 4f shows the test results of the measured output (*I*_out_) under the projections of different letters (a duration of 0.5 s), and it leads to the distinct *I*_out_ during the presentations of different input images. It is observed that the *I*_out_ exceeds the threshold (here is 70 nA) when an image belonging to the target letter (i.e., ‘X’) is presented (Fig. S8b), while it is always lower than the threshold when the images of other classes are presented. Moreover, the measured output currents (*I*_out_) agree well with the theoretically calculated ones. The test results exhibit great performance for image recognition, with a fast response and high SNR (though there still exists the background dark current). The weights exhibit only slight changes after recognition, demonstrating the reliability as a classifier. Apart from that, a 10 × 10 array of the same architecture replaced with resistors are tested for high-speed recognition of digits (Fig. S8c). Projection of different images possesses a duration of 100 μs, and the recognition time is less than 2 μs (Fig. S8d). This is an optoelectronic calculation performed during the conversion of optical signals to electrical signals.

To analyze the potential of complex image recognition, the test of fingerprints with 256 × 256 pixels is carried out here (Fig. S4). The classifier has 65,536 pixels and is used to distinguish the target fingerprint, trained on fingerprint images database. The accuracy of training and test eventually tends to 95% ~100% and 85% ~90%, respectively. This provides a theoretical basis for the hardware implementation of complex recognition. To implement the 256 × 256-pixel array, it is necessary to consider the large-scale integrated scheme where the transistors should be adopted instead of R_0_ to prevent the crosstalk and miniaturize the unit cell (more details in Fig. S11). As well, the CMOS technology can be used in the fabrication for CMOS compatible PDs (such as Si PD, thin film detectors, etc.), while chip bonding can be used to integrate the detector chip with the RRAM array for CMOS incompatible PDs (such as GaSb-based infrared detection).

It is confirmed that the PD-RRAM array could implement MAC operations and in-sensor computing for image recognition. And the accuracy of recognition increases with the number of pixels, that is, scaling the PD-RRAM array up would improve the performance of this architecture. Besides complex image recognition, this design can also be used as filter kernels for convolution^10^. Moreover, there is no electrical crosstalk between pixels here in this array, that is, the output current generated by a pixel would not flow through a neighboring pixel. It can be attributed to its three-terminal design and working under the short-circuit condition.

### Robustness analysis for image recognition

**Fig. 5:**
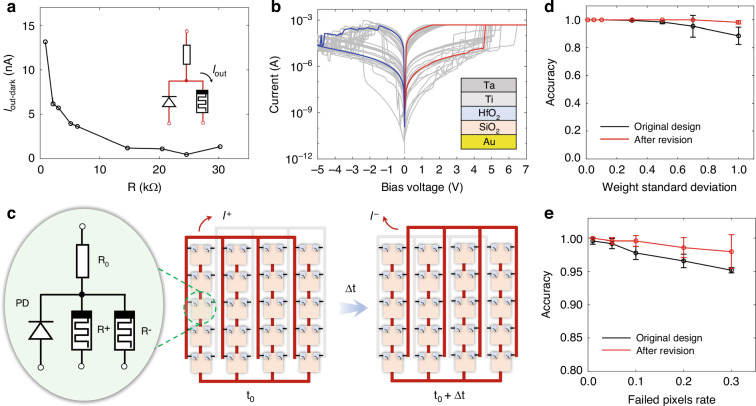
Robustness of the PD-RRAM array for image recognition. **a** The measured dark current of the *I*_out_ under the short-circuit condition. **b** Current–voltage characteristic curve of the redesigned RRAM under DC sweeping voltage between −5 V and 7 V with a SET compliance current (I_CC_) of 500 μA. **c** Schematic illustration of the PD-RRAM array with a differential pair of RRAMs to realize signed weights. The MAC operations of positive weights (R^+^) and negative weights (R^−^) are performed at *t*_*0*_ and ${t_{0}}+\varDelta{t}\,({\varDelta{t}}\,<\,1\,{\upmu{\rm{s}}})$, respectively, and are then subtracted from each other by peripheral circuits. **d** Accuracy of the classifier training for different weight standard deviations. The classifier has 784 pixels and is used to distinguish ‘5’, trained on the MNIST database of handwritten digits. **e** Accuracy of the classifier training for different failed pixel percentages. Here the classifier sets the weights of random pixels to 0, trained as **d**

After verifying the feasibility of this architecture, the robustness of the PD-RRAM array for image recognition is analyzed. As mentioned before, the cell has a dark current of ~10^−^^9^ A and a noise of ~10^−^^11^ A, which may lower the ratio of the effective output current to the dark one. Figure 5a shows that the dark current of the *I*_out_ decreases with the increase of the RRAM resistance, which indicates that it’s mainly determined by RRAMs rather than PDs. And therefore, it is the primary goal to reduce the dark current of RRAMs whose materials and structures need to be optimized. Figure 5b shows an optimized design of RRAMs (Ta/Ti/HfO_2_/SiO_2_/Au), adding a layer of SiO_2_ film that is fabricated by plasma-enhanced chemical vapor deposition (PECVD) and etched by reactive ion etching (RIE). This switching layer, composed of different materials, effectively reduces the dark current of RRAMs to ~10^−^^10^ A (@HRS) and ~10^−^^9^A (@LRS). For this architecture, reducing the dark current and switching voltage has a crucial impact on the overall system performance. In 2017, Yao et al.^50^ reports a structure that balance these parameters more effectively, which suppresses the dark current while moderately reducing the switching voltage.

In addition, using a pair of RRAMs to represent signed weights can also enhance the robustness of systems, and the PD-RRAM array after revision is schematically illustrated in Fig. 5c. A single pixel has a pair of RRAMs and realizes signed weights with a differential pair. The MAC operations of positive weights (*R*^*+*^) and negative weights (*R*^−^) are performed at *t*_*0*_ and *t*_*0*_ + *Δt* (*Δt* < 1 μs) respectively, and the results are subtracted from each other in the peripheral circuits. The final result (*I*) is expressed as

4 $$I={I}_{+}-{I}_{-}$$

where the *I*^*+*^ and *I*^−^ are measured at *t*_*0*_ and *t*_*0*_ + *Δt*, respectively. The signed weights could improve the robustness and stability of this system to a certain extent, reducing the interference of noise and other factors.

Robustness of the PD-RRAM array for the image noise is first analyzed. The fabricated PDs possess a photocurrent standard deviation of less than 5% (Fig. S3a), and thus a different noise level is added in the training/inference (MNIST database shown in Fig. S3b). The classifier has 784 pixels and is used to distinguish ‘5’. Accuracy of the classifier during training in the original design converges slower than that after revision (Fig. S3c, d). As well, the weight values also influence the accuracy of the classifier. Next, robustness of the PD-RRAM array for the weight errors and failed pixels rate is analyzed. According to characteristics of this architecture, the weight fluctuation increases with the decrease of the RRAM resistance (Fig. S9a, b), and different weight standard deviations are added in the inference. Figure 5d shows that the design after revision possesses a stronger anti-noise ability than the original one (Fig. S3e, f). The malfunction of the pixels would also affect the whole performance of the PD-RRAM array. The different failed pixels rates are added in the inference, and weights of some pixels are set to 0 (or 0.5) at random. It shows that failed pixels have a significant impact, as the output is jointly contributed by each pixel (Fig. S9c,d). However, the randomness of failed pixels would also bring different effects on the recognition results. Figure 5e indicates that the design after revision possesses better robustness than the original one. From results, it can be seen that when the rate is controlled within 10%, the accuracy still remains at a high level. See Fig. S9 for more information.

Moreover, this architecture can be designed to implement multi-class recognition and each pixel consists of a PD and *2* *M* RRAM, used for the *M*-class recognition (Fig. S10a). For the reading mode, terminals of the *R*_0_, PD, and corresponding RRAM are under the short-circuit condition, with other terminals floated. A single-layer ANN is used for multi-class recognition in the array, with 20 outputs to distinguish ‘0’–‘9’ of MNIST database (Fig. S10b, c). The accuracy of training and test eventually tends to 95% and 70% (Fig. S10d, e), respectively. In this design, higher integration degree can be achieved than that with each pixel divided into *M* subpixels, due to the smaller size of RRAMs. With the number of RRAMs and pixels increasing, the recognition accuracy and system robustness of this array would be improved. Nevertheless, large scaling of the PD-RRAM array still remains a mainly technological task. The SNR degradation caused by the device miniaturization needs further investigation. The PD-RRAM array in a practical large-scale array chip may be affected by crosstalk, which remains to be studied.

## Discussion

In summary, the PD-RRAM array is presented for in-sensor computing to implement pattern recognition and image pre-processing. The physical network could reduce data conversion and digital signal processing, improving the efficiency of photoelectric signal conversion^51^. This concept provides possibilities for the IoT applications with improved speed, energy efficiency, and reliability. The PD-RRAM unit cell, with characteristics of the reconfigurable optoelectronic output and fast response, provides a device technology foundation for this architecture. The PD-RRAM array is further demonstrated, performing MAC operations between optical images and weights, and can be used to implement real-time image processing functionalities (such as classification). Due to the capability of pixel-level parallel computing, self-powered characteristics in MAC operations, and combination with machine learning, this architecture could achieve high reliability, ultralow latency, and low power consumption for inference. The stable electrical connection between PDs and RRAMs has been proven to be feasible. This work demonstrates a universal design that could be adopted for PDs of various materials, opening up a new way for the hardware implementation of real-time machine vision in different bands. However, this type of physical signal computation for sensing paradigm also has many risks and shortcomings. The SNR of optoelectronic signals will greatly affect the calculation performance (for instance, the accuracy of recognition). The noise issue still needs improvements, though we have achieved great experimental results so far. The noise problem is hard to ignore with the scaling up of the PD-RRAM array. Future work would focus on the monolithic integration and large-scaling of the PD-RRAM array with interconnects and control electronics, which can be implemented with the CMOS technology. There are still many problems to be solved in this process, including the cross-bar structure of large arrays, the uniformity and miniaturization of devices, and the test of large arrays.

## Materials and methods

### Device fabrication

A layer of intrinsic Si is epitaxially grown on a n-Si substrate. Next, B ions are implanted on the surface of intrinsic Si, and a p-i-n junction is formed after thermal annealing. The device patterns are defined through photolithography. Then, the p-Si is etched to form the electrical isolation of pixels by RIE in SF_6_ plasma for a certain time. A 200-nm-thick SiO_2_ layer is then deposited on the surface by plasma-enhanced chemical vapor deposition (PECVD), and the oxide is windowed by RIE in CF_4_ plasma. Contact electrodes (Al/Au = 20/100 nm) are formed by a lift-off process using magnetron sputtering, with thermal annealing at 350 °C and N_2_ atmosphere to form ohmic contacts. Again, a 100-nm-thick SiO_2_ layer is deposited on the surface by PECVD, and the oxide is etched by RIE to form holes. Then, the 100-nm-thick NiCr alloy is sputtered as R_0_, formed by a lift-off process. Next, an 8-nm-thick HfO_2_ layer is deposited using atomic layer deposition (ALD) at 250 °C, and is etched by inductively coupled plasma in Ar plasma. At last, top electrodes of RRAMs (Ti/Ta/Au = 5/50/100 nm) are formed by a lift-off process using magnetron sputtering.

There are still significant difficulties and challenges, including: (1) The processing technology of large-scale arrays; (2) The uniformity and production yield of devices within the array; (3) The noise and dark current in the array. The large-scaling of the array is closely related to the stability and uniformity of devices. The devices in this design mainly include PDs and RRAMs, with the former possessing a consistency of 1% ~5% and the latter having relatively poor uniformity. Due to the fact that the production yield of devices has not reached 100%, the higher the number of pixels in the array, the more likely it is to have bad pixels in the array. Accordingly, the uniformity and production yield are significant factors that cannot be ignored if the array is scaled up. In order to achieve more stable and uniform devices, the material and structure of the oxide layer are crucial. Here, the hole structure is adopted for RRAMs, and the HfO_2_ made by ALD could provide a more uniform and denser layer, which also lowers the noise and dark current for this array.

### Measurements

The *I*–*V* characteristics are measured with Agilent/HP 4155 C. The pulse measurements are implemented by Keysight 1500 A. In the photovoltaic measurement, a LED with tunable light intensities is used as the light source. Only the windows of PDs are considered as being subjected to the illumination and used for the calculation of light power. The LED light is spatially modulated by a DMD (ViaLUX).

### Experimental setup

Schematics of the experimental setup are shown in Fig. 4e. The LED light is spatially modulated by a DMD (ViaLUX). The letters are displayed with a frame frequency of ~2 fps. The resulting image is projected onto the 3 × 3 PD-RRAM array chip using lens. The PD-RRAM array chip is programmed into theoretical weight matrixes by source meters (Keysight 1500 A and Agilent 4155 C). For time-resolved measurements, the output current is recorded with Agilent 4155 C.

## Supplementary information

Supplementary information for Optoelectronic array of photodiodes integrated with RRAMs for energy-efficient 1 in-sensor computing

## Data Availability

Source data are provided in this paper. The data that supports the other findings of this study are available from the corresponding authors upon reasonable request.
