# Supplementary material for: Optoelectronic array of photodiodes integrated with RRAMs for energy-efficient in-sensor computing
Source: Light Sci Appl. 2025 Jan 15;14:48. doi: 10.1038/s41377-025-01743-y (PMC11736068; doi:10.1038/s41377-025-01743-y)
Supplement: Supplementary file 1 — Supplementary information for Optoelectronic array of photodiodes integrated with RRAMs for energy-efficient 1 in-sensor computing [file 41377_2025_1743_MOESM1_ESM.docx]

**Supplementary Information for “Optoelectronic array of photodiodes integrated with RRAMs for energy-efficient in-sensor computing”**

*By*

*Wen Pan^1^*

*Lai Wang^2^**

*Jianshi Tang^3^*

*Heyi Huang^3^*

*Zhibiao Hao^2^*

*Changzheng Sun^2^*

*Bing Xiong^2^*

*Jian Wang^2^*

*Yanjun Han^2^*

*Hongtao Li^2^*

*Lin Gan^2^*

*Yi Luo^2^*

October 23, 2023

^1^ W. Pan is affiliated with Department of Electronic Engineering, Tsinghua University. ^2^ Prof. L. Wang, Prof. Z. Hao, Prof. C. Sun, Prof. B. Xiong, Dr. J. Wang, Dr. Y. Han, Dr. H. Li, Dr. L. Gan, Prof. Y. Luo are affiliated with Beijing National Research Center for Information Science and Technology (BNRist) and Department of Electronic Engineering, Tsinghua University, Beijing 100084, China. ^3^ Prof. J. Tang, Dr. H. Huang are affiliated with School of Integrated Circuits, Tsinghua University, Beijing 100084, China.

*Correspondence to Lai Wang (e-mail: wanglai@tsinghua.edu.cn).

**Optoelectronic array of photodiodes integrated with RRAMs for energy-efficient in-sensor computing**

*Wen Pan, Lai Wang*, Jianshi Tang, Heyi Huang, Zhibiao Hao, Changzheng Sun, Bing Xiong, Jian Wang, Yanjun Han, Hongtao Li, Lin Gan, Yi Luo*

**Supplementary information**


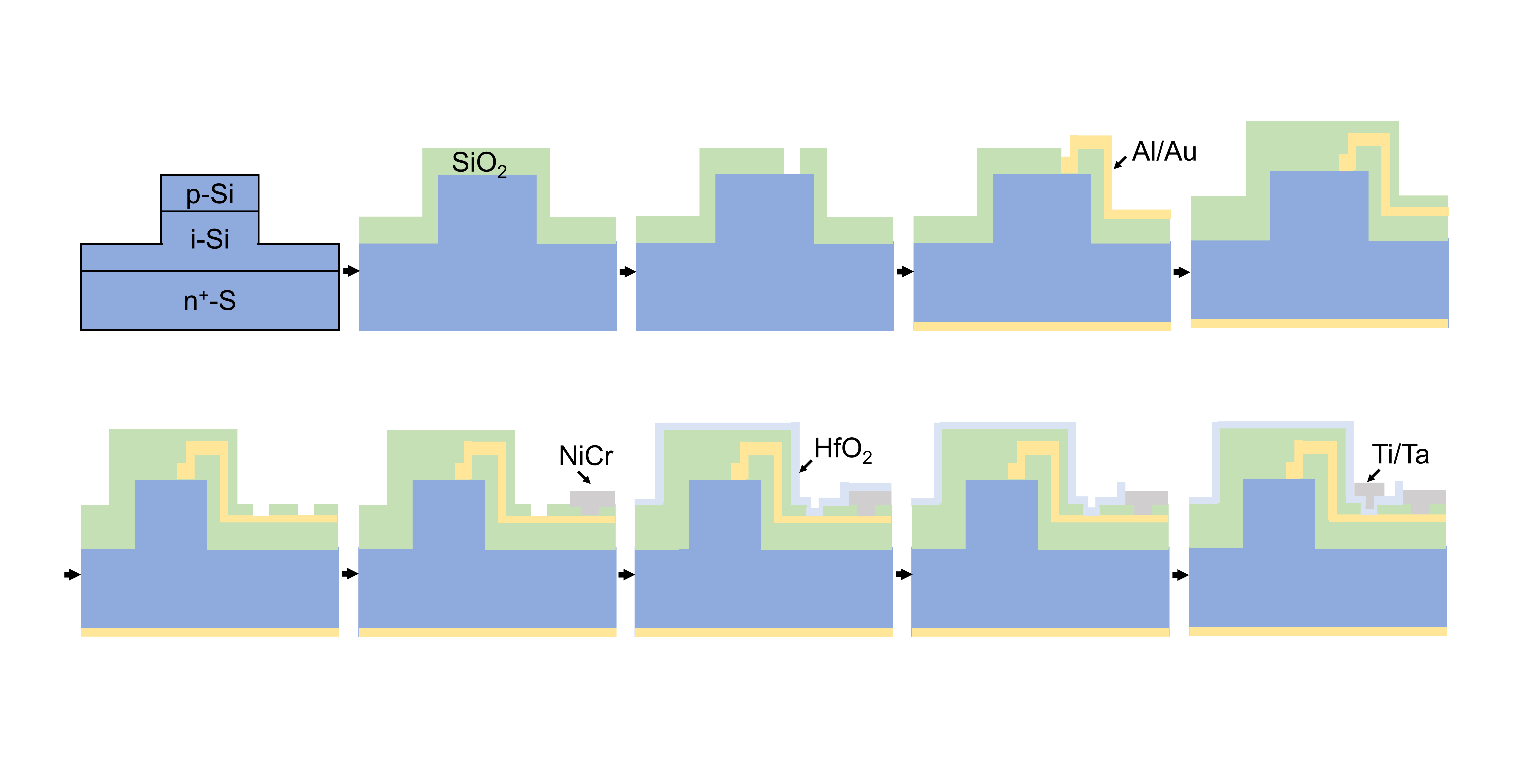


**Supplementary Figure 1.** Cross-sectional schematic illustration of fabrication steps for the PD-RRAM device.

A layer of intrinsic Si is epitaxially grown on a n-Si substrate. Next, B ions are implanted on the surface of intrinsic Si, and a p-i-n junction is formed after thermal annealing. The device patterns are defined through photolithography. Then, the p-Si is etched to form the electrical isolation of pixels by reactive ion etching (RIE) in SF_6_ plasma for a certain time. A 200-nm-thick SiO_2_ layer is then deposited on the surface by plasma-enhanced chemical vapor deposition (PECVD), and the oxide is etched by RIE in CF_4_ plasma. Contact electrodes (Al/Au = 20/100 nm) are formed by a lift-off process using magnetron sputtering, with thermal annealing at 350 ℃ and N_2_ atmosphere to form ohmic contacts. Thus, Si-PDs have been formed. Again, a 100-nm-thick SiO_2_ layer is deposited on the surface by PECVD, and the oxide is etched by RIE to form a hole-structure. By adopting a hole-structure, the surface is more suitable for preparing RRAMs. Then, the 100-nm-thick NiCr alloy is sputtered as R_0_, formed by a lift-off process. Next, an 8-nm-thick HfO_2_ layer is deposited using atomic layer deposition (ALD) at 250 °C, and is etched by inductively coupled plasma (ICP) in Ar plasma. At last, top electrodes of RRAMs (Ti/Ta/Au = 5/50/100 nm) are formed by a lift-off process using magnetron sputtering. Therefore, the MIM structure of RRAMs is composed of Au as the bottom inert electrode, and Ti as the top active electrode.

The consecutive direct current (D.C.) write/erase behaviors on switching characteristics of an individual RRAM with a compliance current (*I_CC_*) of 100 μA in the SET process and a stop voltage of −2.5 V in the RESET process. With a typical SET or RESET pulse train of 10 ns applied to the RRAM, the resistance switches from 1 kΩ to 100 kΩ with at least 10 resistance states.


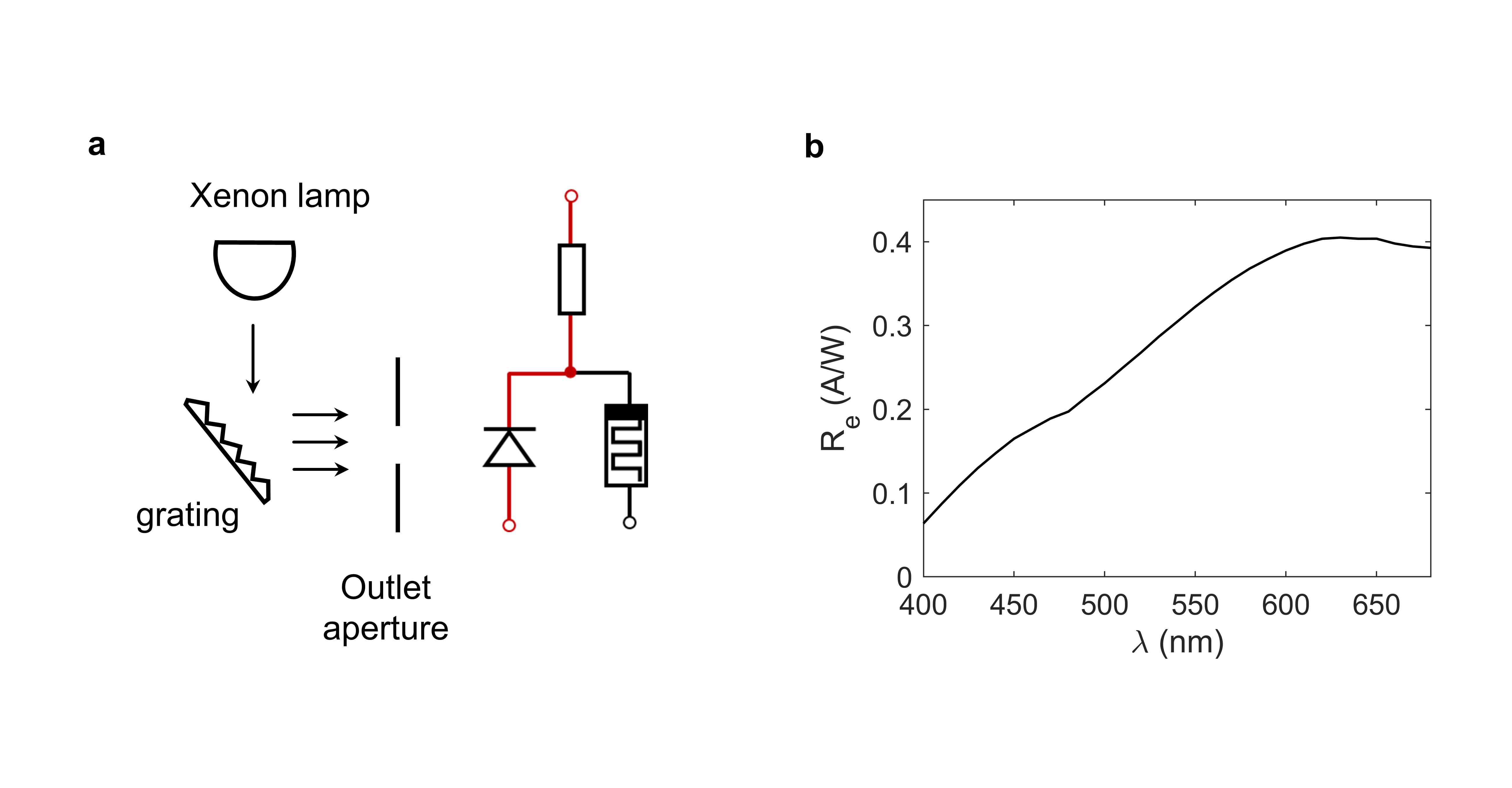


**Supplementary Figure 2.** Spectral response measurement of Si-PDs. **a,** Schematic illustration of the optical setup for spectral response measurements. A xenon lamp is used as the light source, split by gratings to output the monochromatic light (*Δλ* = 1 nm). **b,** Measured spectral response of Si-PDs. The *λ* is stepped up from 400 nm to 680 nm with a 10 nm step, with zero bias voltage applied to PD.


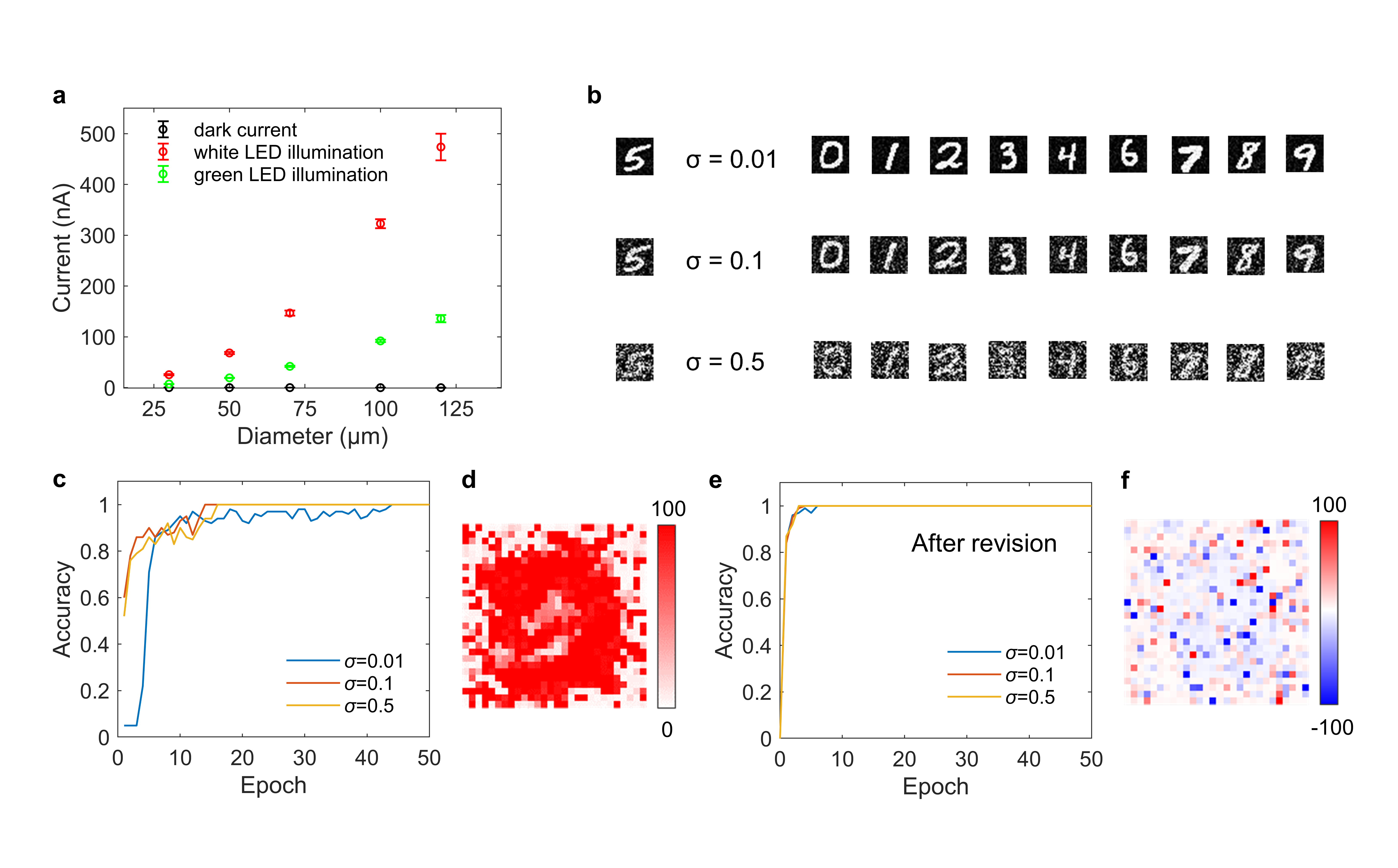


**Supplementary Figure 3.** Robustness analysis of the PD-RRAM array network for image noise. **a,** Photocurrent standard deviation of PDs for varying diameters (10 devices each) and varying light wavelength. The photocurrent standard deviation of PDs is in the range of 2%-5%. **b,** Datasets with a noise level (*σ*) of 0.01, 0.1, 0.5 respectively. The classifier has 784 pixels and is used to distinguish ‘5’, trained on the MNIST database of handwritten digits. **c,** Accuracy of the classifier during training for varying artificial noise levels in the original design. **d,** Theoretical RRAM resistances (unit: kΩ) in the original design. The RRAM resistance ranges from 1 kΩ to 100 kΩ. **e,** Accuracy of the classifier during training for varying artificial noise levels after revision. **f,** Theoretical RRAM resistances (unit: kΩ) after revision. The RRAM resistance ranges from 1 kΩ to 100 kΩ, and -1 kΩ to -100 kΩ.


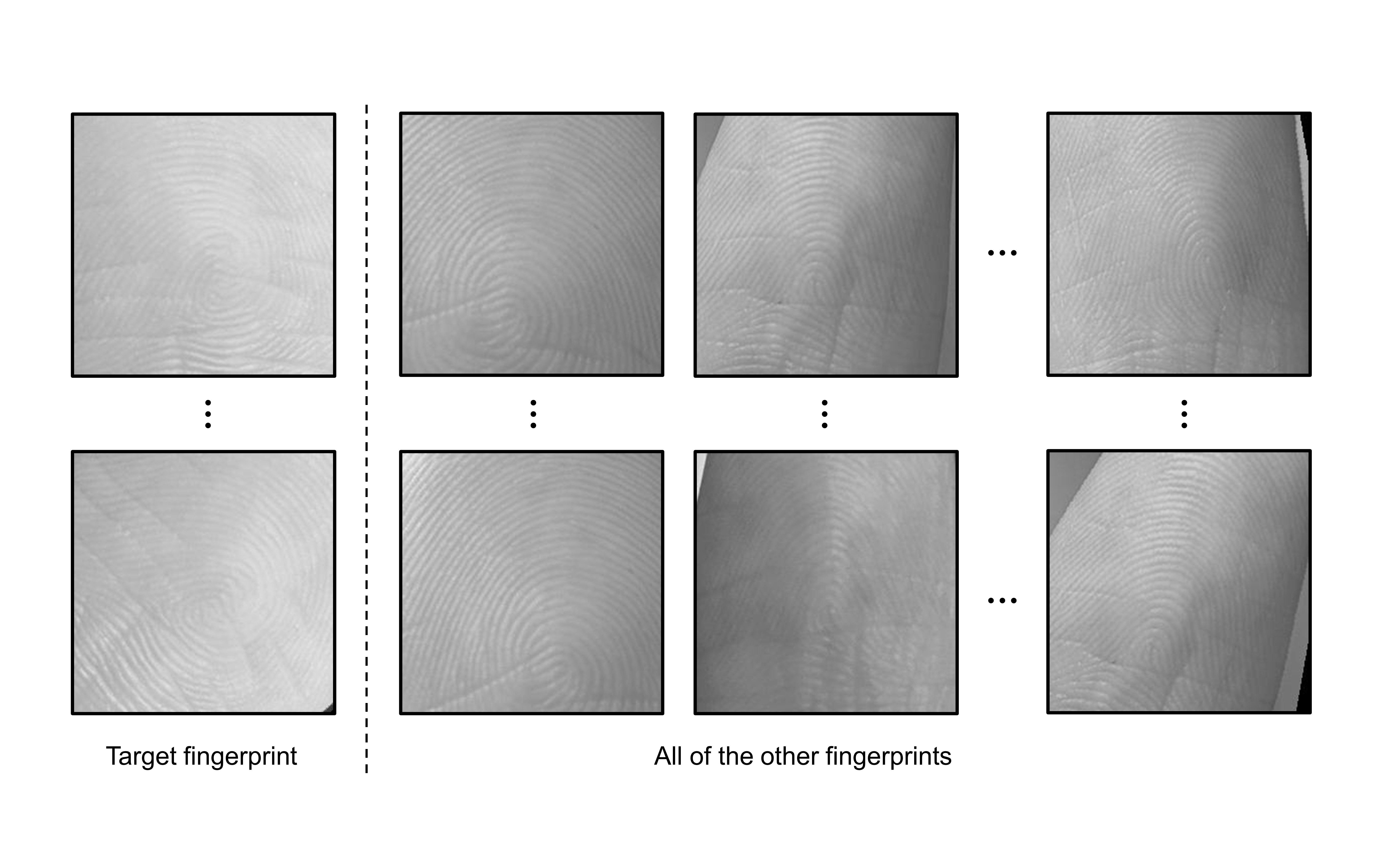


**Supplementary Figure 4.** The recognition test of the PD-RRAM array for complex images. The training task is to identify the target fingerprint.

The classifier has 65,536 pixels and is used to distinguish the target fingerprint, trained on the database of fingerprints. 39 and 20 fingerprint images are used for training and test respectively, collected by cameras. The model used here is the artificial neural network (ANN), i.e. the fully connected layer. The size of the fully connected layer matrix is 65536 × 2. The noise level (*σ*) here is 0.05. The accuracy of training and test eventually tends to 95% ~ 100% and 85% ~ 90% respectively.


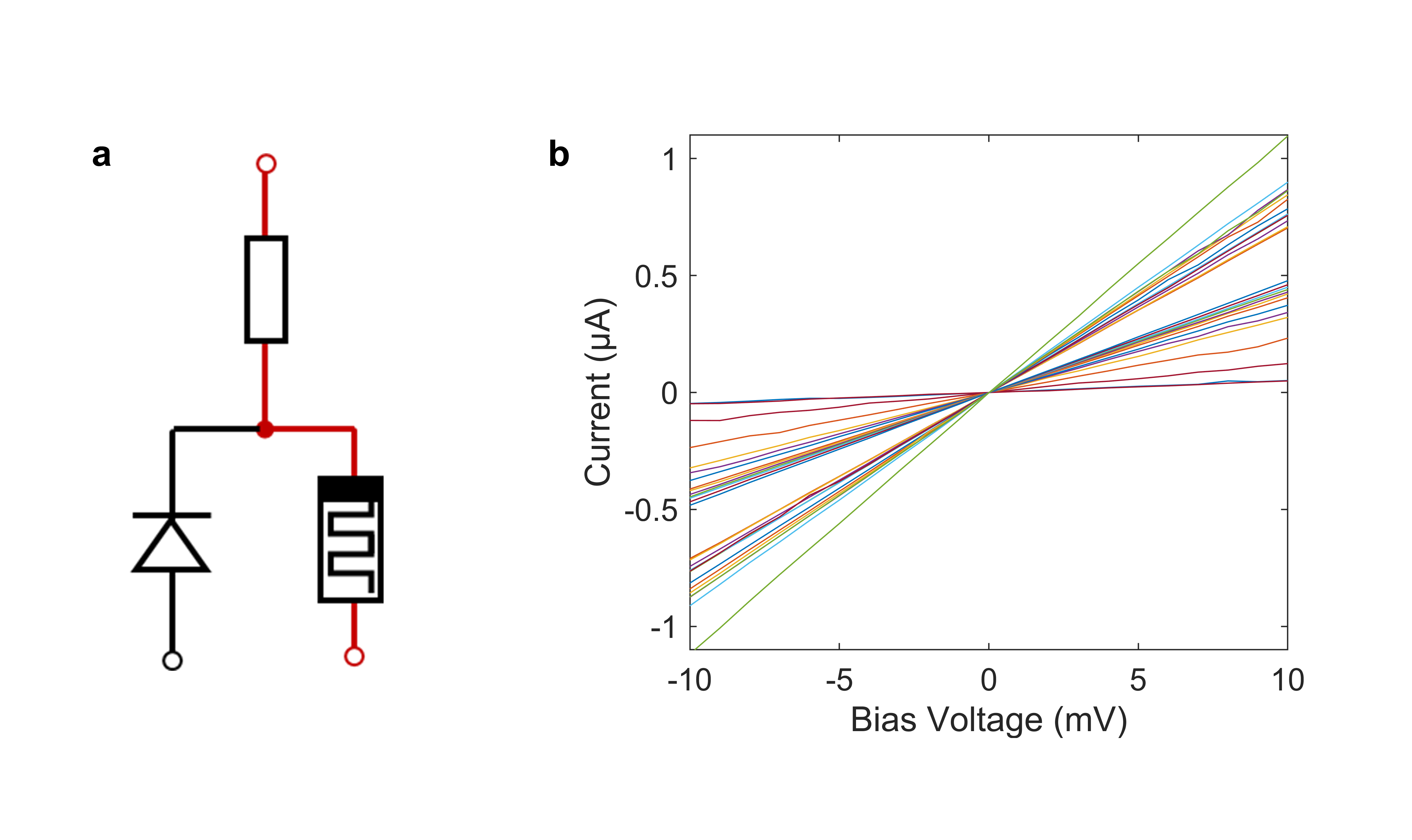


**Supplementary Figure 5.** Multi-resistance states of RRAMs. **a,** Writing Mode of the PD-RRAM cell. **b,** Current–voltage characteristic curve of the RRAM. An individual memristor is tuned into multi-level resistance states by high-resolution off-chip driving circuitry, and each resistance level is read by a D.C. voltage sweeping from -10 mV to 10 mV.


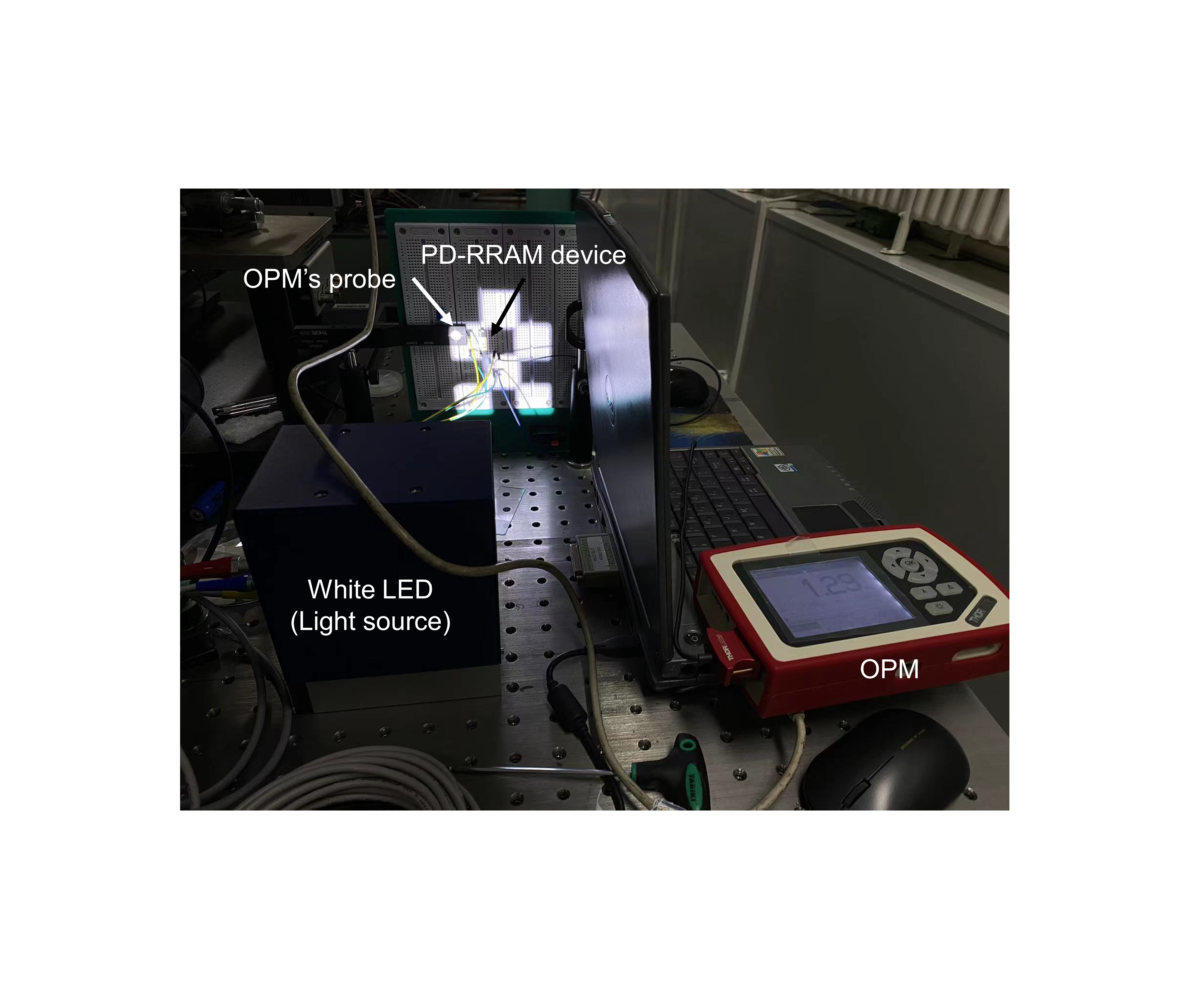


**Supplementary Figure 6.** Photograph of the optical setup in Fig. 2d.


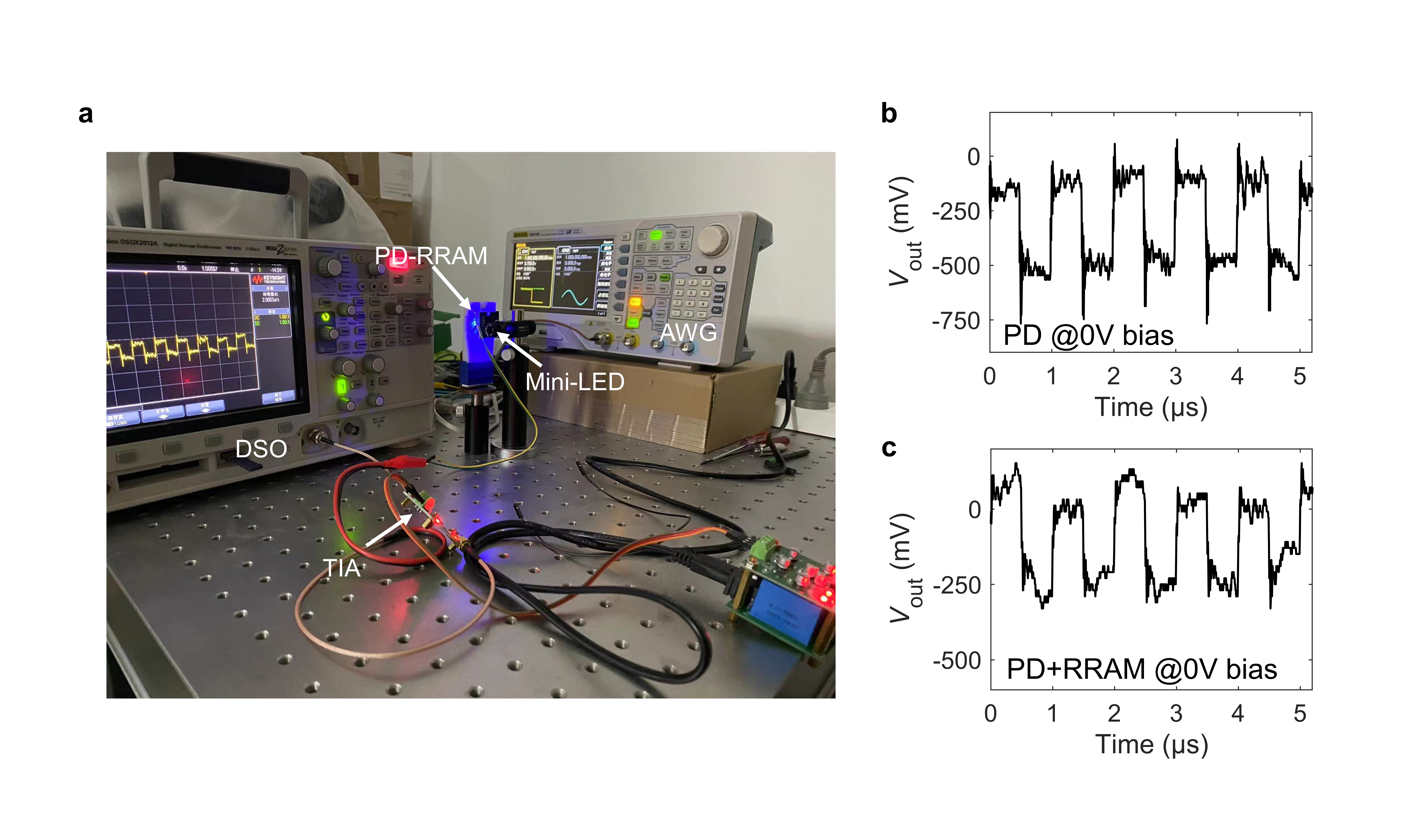


**Supplementary Figure 7.** Response measurement of the PD-RRAM cell. **a,** Photograph of the optical setup in Fig. 3b. **b,** Response of the PD with the pulsed light under the short-circuit condition. Rise and fall times of a pulse, measured between 10% and 90% of the full pulse amplitude, are both 25 ns. **c,** Output response of the PD-RRAM cell with pulsed light under the short-circuit condition. Rise and fall times of a pulse, measured between 10% and 90% of the full pulse amplitude, are both 30 ns.


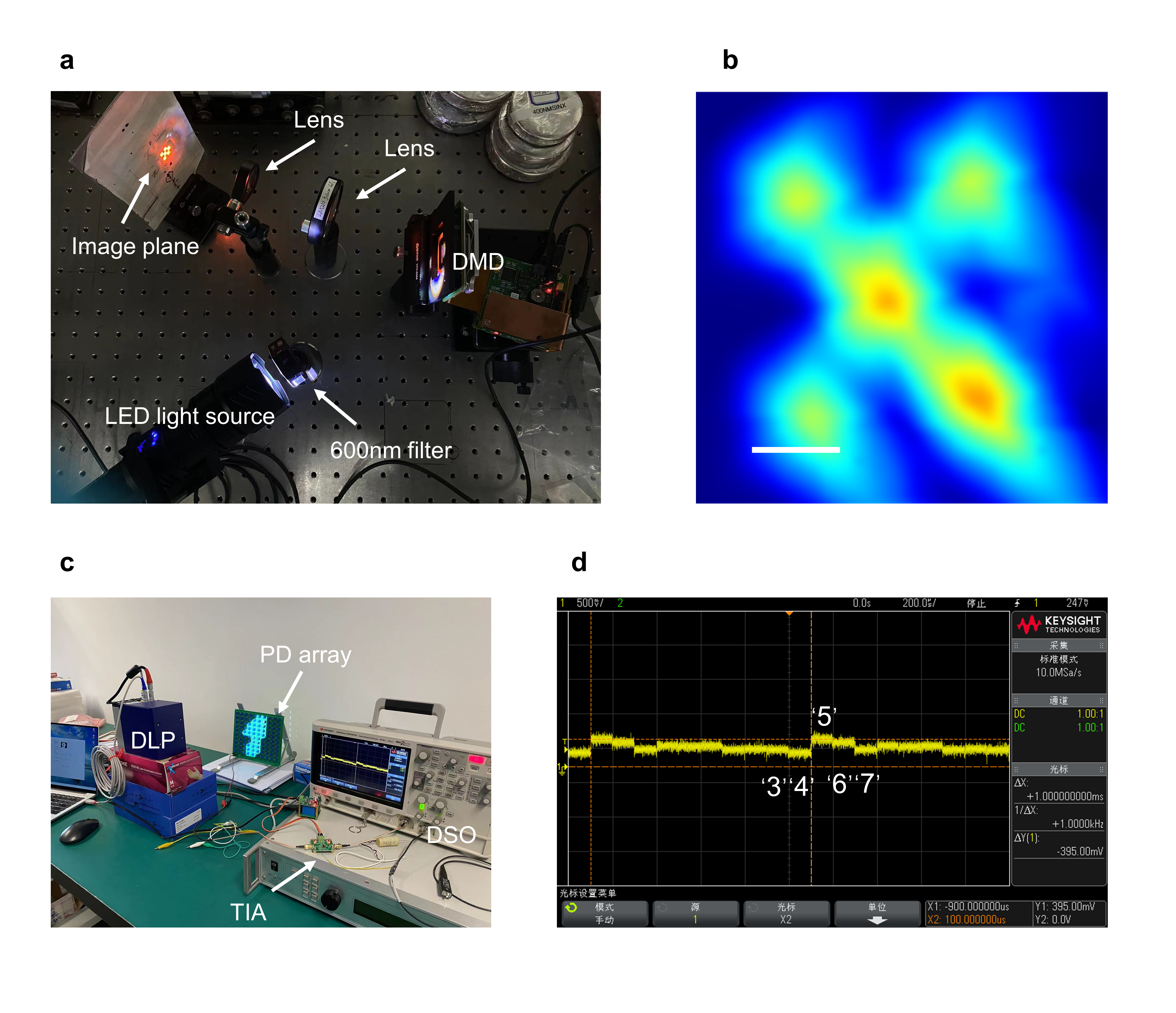


**Supplementary Figure 8.** Image recognition test. **a,** Photograph of the optical setup in Fig. 4e. The DMD is used for spatial light modulation. **b,** Optical pattern of ‘X’ photographed by a charge coupled devices (CCD) in the image plane. Scale bar, 300 μm. **c,** Photograph of the optical setup. Replaced with resistors, a 10×10-pixel array of the same architecture is tested for high speed recognition. A digital light processing (DLP) is used to project images of digits. **d,** Measured output for recognition. Projection of different digits ‘0’ - ‘9’ possesses a duration of 100 μs, and the classifier is used to distinguish ‘5’. The recognition time is less than 2 μs and the corresponding frame rate is 0.5 Mfps, which is limited by the speed of the DLP.


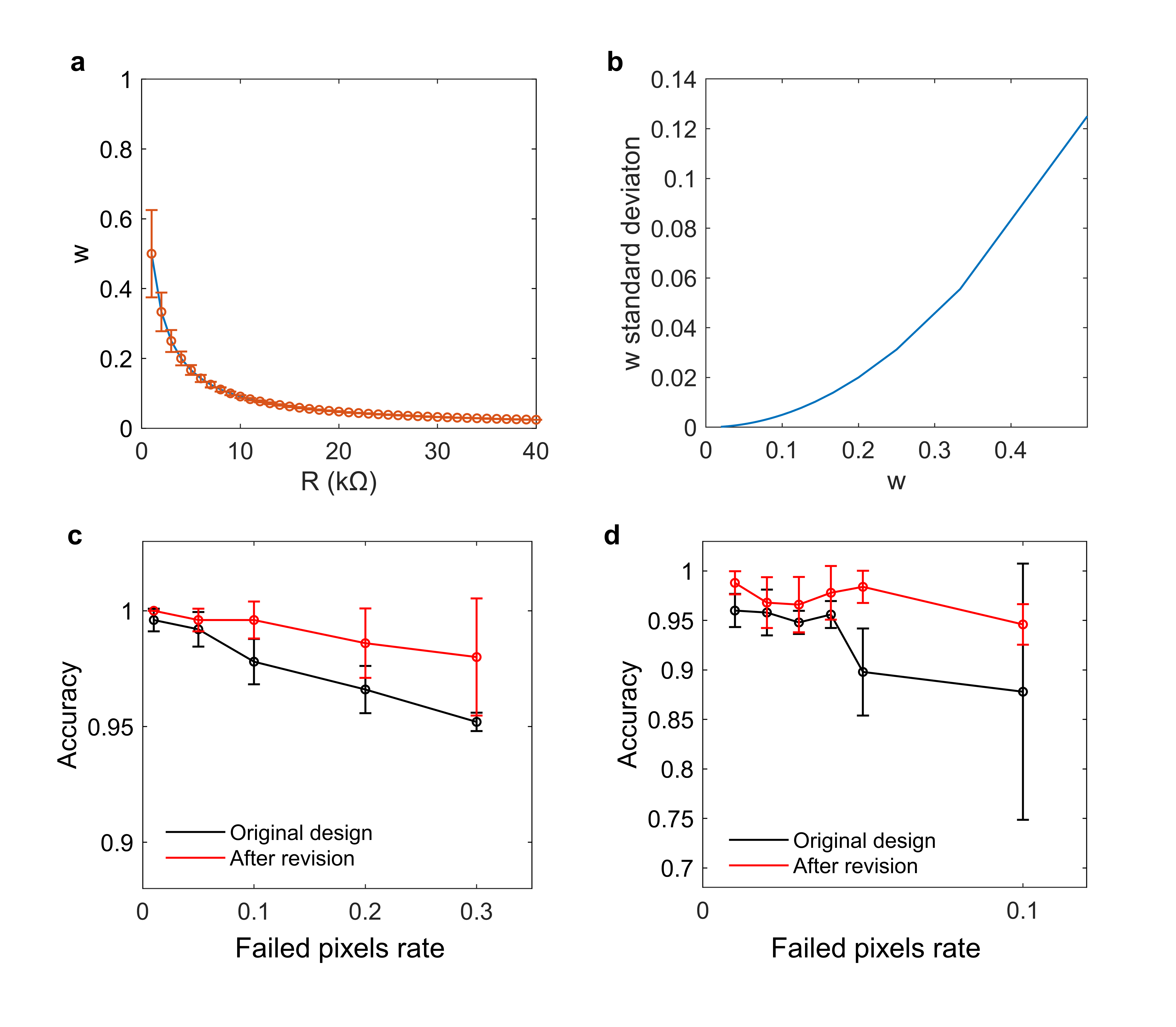


**Supplementary Figure 9.** Robustness test of the PD-RRAM array for weight errors and failed pixels. **a,** The relationship between the RRAM resistance state and actual weight. According to characteristics of the architecture, the weight fluctuation increases with the decrease of the RRAM resistance. **b,** The relationship between the weight and standard deviation. The weight fluctuation increases with weights. **c,** Accuracy of the classifier training for varying failed pixels rates (set to 0). The classifier sets the weights of some pixels to 0 at random, trained as Fig. S3b. **d,** Accuracy of the classifier training for varying failed pixels rates (set to 0.5). The classifier sets the weights of some pixels to 0.5 at random, trained as Fig. S3b.


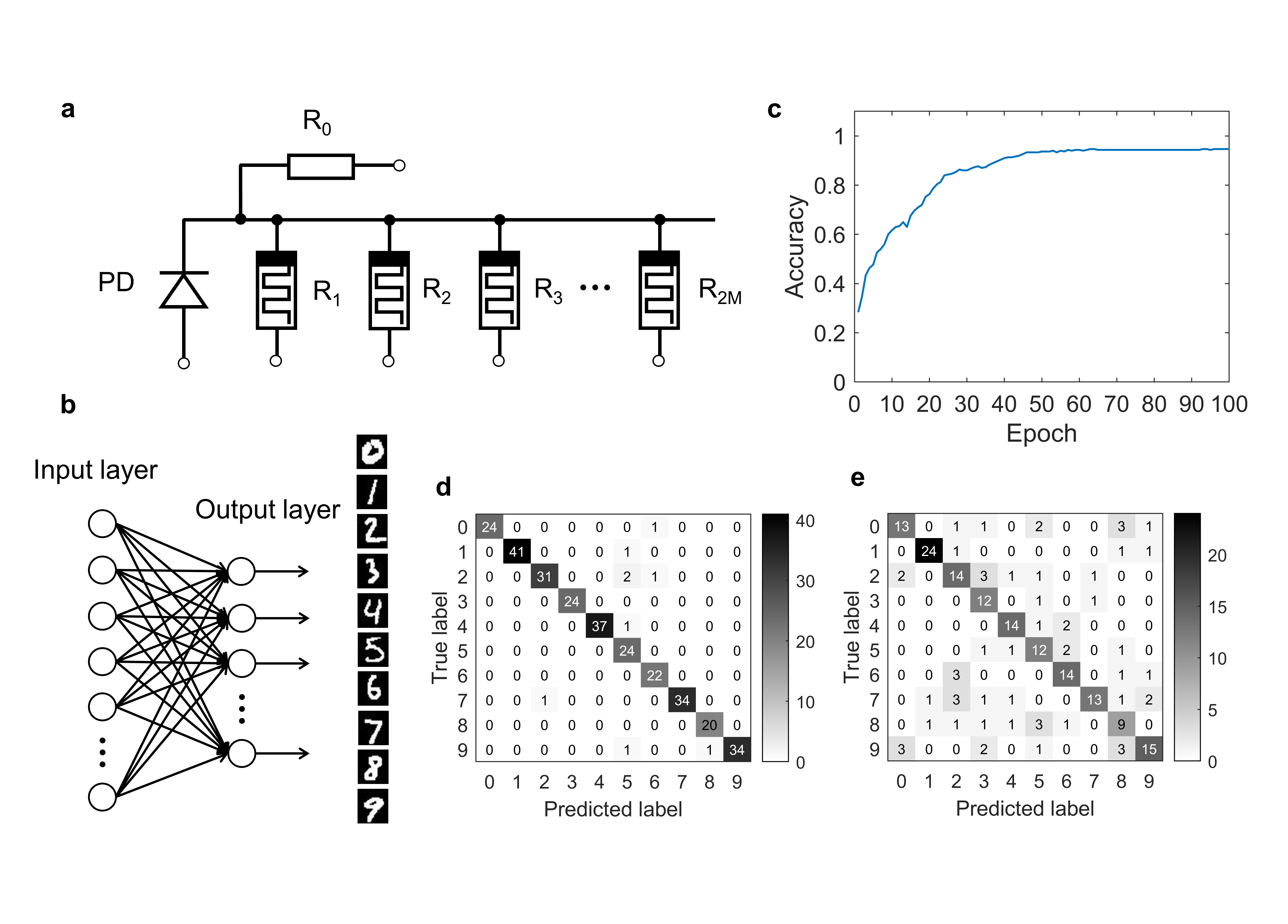


**Supplementary Figure 10.** Multi-class recognition design. **a,** Schematic illustration of the PD-RRAM array networks for multi-class recognition. Each pixel consists of a PD and *2M* RRAMs, used for *M*-class recognition. Detecting mode: control terminals of the R_0_ and PD with the RRAMs’ terminals suspended. Writing mode: control terminals of the R_0_ and corresponding RRAM. Reading mode: control terminals of the R_0_, PD and corresponding RRAM under the short-circuit condition. **b,** A single-layer ANN used for multi-class recognition in the PD-RRAM array. The classifier has 784 pixels and 20 outputs, is used to distinguish ‘0’ - ‘9’. **c,** Accuracy of the classifier during training, trained on the MNIST database of handwritten digits. The accuracy of training and test eventually tends to 95% and 70% respectively. **d,** Confusion matrixes of training for the PD-RRAM array. **e,** Confusion matrixes of the test for PD-RRAM array.


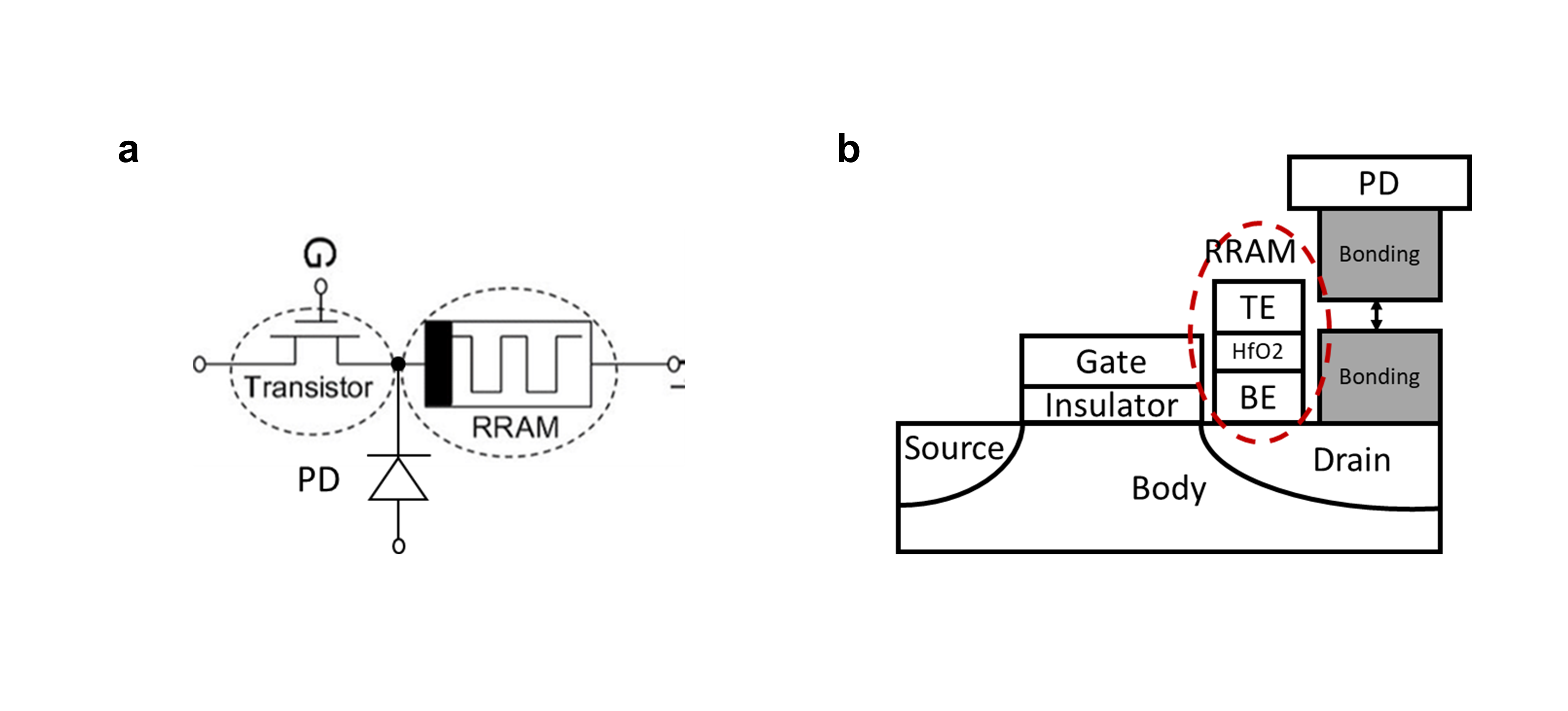


**Supplementary Figure 11.** Large-scale integration scheme. **a,** Array units for the large-scale integration and device minimization. **b,** Integrated scheme for photodiodes that are incompatible with CMOS technology.

Crosstalk analysis: As for the detecting mode and reading mode, the crosstalk has little impact because the polarity of PDs avoids the flow of currents within this array. Crosstalk mainly occurs in the writing mode, where the bidirectional conductivity of RRAM makes it much more likely to occur. Assuming that the leakage current of the MOS is ~ 1nA @*V_d_* = 1V (i.e. *R_0_* ~ 10^9^ Ω), when a cell is selected for writing in an N×N array where each cell is the same, its current is *I = V/(R) + (N-1)^2^ × V/(3R+2R_0_)*. As *R* is 10^3^ ~ 10^5^ Ω (<< *R_0_*), *I = V/(R) + (N-1)^2^ × V/(2R_0_)*. When *N* is ~ 100, the accuracy is still relatively high (~ 95.24%).
